# The Effectiveness of Multicomponent Intervention on Daily Functioning among the Community-Dwelling Elderly: A Systematic Review

**DOI:** 10.3390/ijerph19127483

**Published:** 2022-06-18

**Authors:** Myeongshin Kim, Eunyoung Shin, Seyoon Kim, Sohyune Sok

**Affiliations:** 1 Department of Nursing, Graduate School, Kyung Hee University, Seoul 02447, Korea; myeongshin@khu.ac.kr (M.K.); ssamyun@khu.ac.kr (S.K.); 2 College of Nursing Science, Kyung Hee University, Seoul 02447, Korea; eyshin@khu.ac.kr

**Keywords:** activities of daily living, aged

## Abstract

The deterioration of physical and cognitive functioning in the elderly is an impairment to their independent self-management and to improving their ability to perform daily functions. Nurses should support the elderly to experience a healthy and a successful aging process by preventing dependence on daily functioning and understanding the care assistance that such persons need. This study aimed to gain insight into the evidence on the effectiveness of multicomponent intervention on the activities of daily living (ADL) and instrumental activities of daily living (IADL) among the community-dwelling elderly without cognitive impairment. The design is a systematic review of a randomized controlled trial. The language of the published literature was English, and the search period was from January 2000 to December 2020. Articles were included under the PICO (population, intervention, comparison, and outcome) framework for: (a) community-dwelling elderly without cognitive impairment; (b) multicomponent intervention; (c) comparison group who did not receive the intervention; and (d) measurement of the effect of ADL and IADL. A total of 4413 references were found, 6 studies were included. Most studies (n = 5) reported that the multicomponent intervention exerted a beneficial effect on ADL and IADL. Only one study showed the highest methodology and reporting quality in the Cochrane review. Common components of the programs included: occupational therapy, physical therapy, exercise, memory training, cognitive–behavioral therapy, interdisciplinary intervention, and cognitive training. Multicomponent intervention may be a beneficial way to improve dependence on ADL and IADL as an important area of functional evaluation in the elderly. Considering the physical condition of the elderly, multicomponent interventions, including physical activity, exercise, occupational therapy, and especially individually customized coaching related to ADL and IADL training, may be useful.

## 1. Introduction

Population aging is directly linked to important public health and public policy issues. Globally, the population growth rate was 6% for those aged 65 years and older in 2019, but it is projected to increase to 16% by 2050 [1]. The prolongation of life expectancy and the increase in the elderly population are remarkable in many countries around the world, resulting in population aging that can lead to an increase in age-related dysfunction and cognitive dysfunction [2,3]. Due to the slowing of activities according to the weakening of physical and mental energy [3,4], health-seeking behaviors, including health care, are also restricted. As such, aging tends to increase the functional dependence of the elderly in daily life due to deterioration of physical and physiological functions and a decrease in economic and social activities [5,6]. Accordingly, in order to maintain healthy aging among the elderly, it is important to address the dependency problem and to support the improvement of functional ability [7,8].

Activities of daily living (ADL), which evaluate the minimum ability for independent living, includes basic activities such as bathing, eating, dressing, toileting, and moving [9]. Instrumental activities of daily living (IADL) consist of complex activities that require more advanced physical and mental activities and skills, such as food preparation, shopping, money management, and housework [10]. ADL and IADL are essential prerequisites for healthy aging, and the maintenance of independent functional ability is the most important factor in quality of life in old age [11]. Therefore, it is necessary to identify behaviors that can prevent and manage functional disorders by improving self-management.

In contrast to single interventions that rely on single-target approaches, such as physical activity or cognitive stimulation, multicomponent interventions are multicomponent approaches that combine two or more intervention strategies [12]. It was emphasized that multicomponent interventions, including strength, mobility, gait, balance, and physical performance, are more effective strategies to improve the physical functioning of the elderly than mere single interventions [13]. In particular, multicomponent interventions, including physical activity, nutrition, vascular management, cognitive training, and social activities, were shown to prevent impairment of ADL and IADL that maintain daily function [14]. As such, it means that multicomponent interventions can have a greater effect by providing a wider range of positive outcomes [12,15]. Therefore, the main purpose of this systematic review is to identify, evaluate, and synthesize the evidence on the effects of multicomponent interventions on the daily functioning of the elderly. Accordingly, nursing care for the elderly can contribute to preventing dependent activities and improving daily functions by considering the functional aspects of the elderly population, as it is the fastest growing group in the field of health care. The purpose of this study is to analyze the characteristics of multicomponent interventions applied to the community-dwelling elderly and to confirm the effect on ADL and IADL.

## 2. Materials and Methods

### 2.1. Study Design

This study is a systematic review that was conducted based on the research guidelines presented by the Cochrane Handbook for Systematic Reviews of Interventions and the framework of PICO (population, intervention, comparison, and outcome) [16].

### 2.2. Search Strategy

Databases used included PubMed, Cochrane Library, EMbase, ProQuest, and MEDLINE. The search terms were “Elderly” [MeSH] OR “Older” [MeSH] OR “Older Adult” [MeSH] OR “Aged” [MeSH]) AND (“Community” [MeSH] OR community-dwelling OR home-dwelling OR community-living OR community-based) AND (“Activities of Daily Living” [MeSH] OR ADL OR instrumental activities of daily living OR IADL) AND (multi component OR multi-component OR multicomponent. The language of the published literature was English, and the search period was from January 2000 to December 2020. Since the 2000s, research has been actively conducted as the importance of multicomponent intervention strategies has been emphasized away from the framework of single intervention, this study has a limit in the search period.

### 2.3. Selection Criteria

In order to review the literature, the literature was selected according to the following criteria.

1. Population: community-dwelling elderly without cognitive impairment
2. Intervention: Multicomponent intervention
3. Comparison: received usual care or different care interventions
4. Outcome: ADL (activities of daily living), IADL (instrumental activities of daily living)
5. Study design: Randomized controlled trials (RCT).

### 2.4. Study Selection

**Figure 1:**
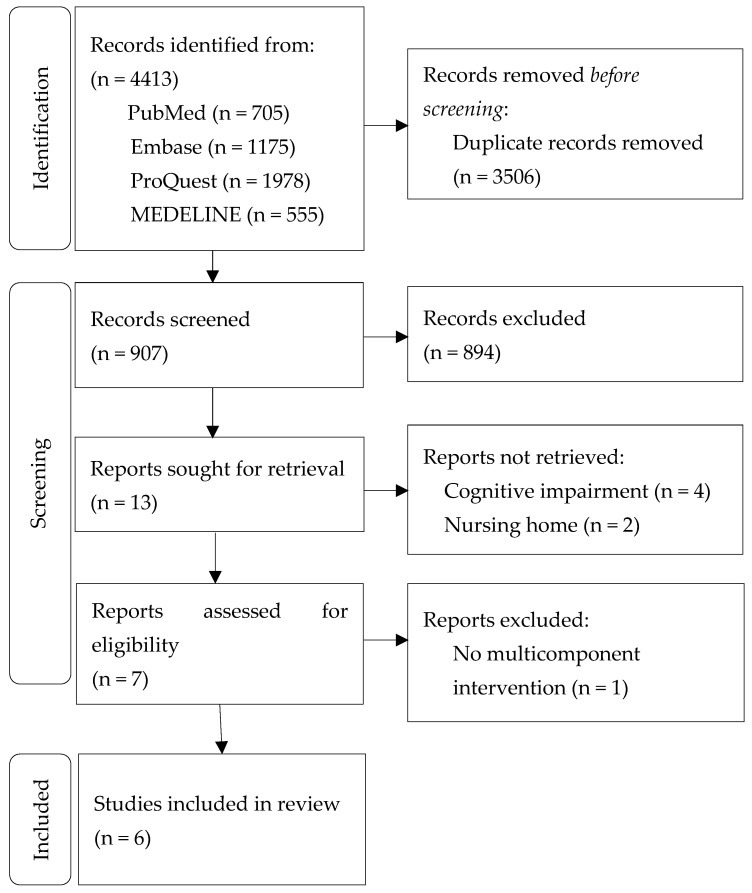
PRISMA (Preferred Reporting Items for Systematic Reviews and Meta-Analyses) flow diagram of the searches and the selection process.

A total of 4413 articles were searched by entering a search term in each database, and 907 articles were selected, excluding duplicate articles. Based on titles and abstracts, 13 articles were selected. They were reviewed again whether or not they met the inclusion criteria, and 4 articles—including subjects with cognitive impairment, and 2 articles, including subjects admitted to nursing homes—were excluded. Of the 7 articles, one article that was not a multicomponent intervention was excluded, and 6 articles were finally included in the review (Figure 1).

### 2.5. Assessing Risk of Bias

The risk of bias in each literature was independently evaluated by four reviewers, and if there was a difference in opinion, it was finally decided through discussion. The Cochrane Collaboration tool [17] was used to assess the main risk in the following seven areas of bias in randomized controlled trials: random sequence generation, allocation concealment, blinding of participants and researchers, blinding of outcome assessment, incomplete outcome data, selective reporting, and other bias. The bias was evaluated as ‘yes’, ‘no’, and ‘unclear’ for each area, and the reviewer judged and evaluated the detailed criteria for yes, no, and unclear in each of the seven areas. ‘Yes’ means low-risk bias, ‘no’ means high-risk bias, and ‘unclear’ means there is insufficient information.

### 2.6. Data Abstraction and Quality Appraisal

The entire process of literature selection was independently performed by four researchers, and in case of disagreement, the final articles were decided through a consultation with a third-party expert. Six articles, which were included based on the inclusion criteria, were independently abstracted in an Excel file by four reviewers.

### 2.7. Synthesis

Specific information for data abstraction included demographics (age and country of origin), study design, theoretical background, evidence of sample size, population, intervention, measurement, and result. Finally, due to the limited number of selected literature and the variability of measurement instruments, meta-analysis or statistical analysis, including an explanation of the effect size, could not be performed.

### 2.8. Ethical Consideration

This study was conducted after obtaining approval from the Kyung Hee University Institutional Review Board (IRB No. KHSIRB-20-193 [RA]).

## 3. Results

### 3.1. Study Characteristics

Table 1 outlines the sample characteristics and methods of the six included studies [18,19,20,21,22,23]. Among the six articles, one was published in 2006, one in 2011, three in 2018, and one in 2020. The countries where the studies were conducted were the United States (two articles), Malaysia (one article), China (one article), the Netherlands (one article), and Thailand (one article). In the selected articles, the theoretical background of the program was reported only in three articles [18,21,22]. The number of subjects who participated in the intervention varied from 12 to 160 (total n = 1102), and the average age was 62.9 to 79.5 years. The rationale for calculating the sample size was reported only in three articles [18,21,22]. The internal consistency related to the reliability of the measurement instrument appeared in only one article [18].

### 3.2. Risk of Bias

The methodological quality of the studies included in this systematic review is presented in Table 2. As a result of the methodological quality evaluation, only one article met all criteria in seven areas [21]. The details of the randomization of subjects were not mentioned in three articles, and they could not be determined [19,20,23]. The concealment of the assignment order was evaluated as a high risk of bias in one article, as participants were informed of the group assigned to them [22]. Double-blinding was evaluated as a high risk of bias in one article, as participants knew the group to which they were assigned [22]. Blinding of outcome assessment was evaluated as high risk of bias, as the intervention staff adjusted the components according to the characteristics of the participants in one article [19]. In terms of incomplete outcome data, one article was evaluated to have a high risk of bias, as it did not mention the reasons of the participants for dropping out [23]. Selective reporting was evaluated as low risk of bias in all articles [18,19,20,21,22,23]. As for other biases, two articles did not mention whether or not the protocol was registered, so it was not possible to judge their appropriateness [18,20].

### 3.3. Intervention Characteristics

As shown in Table 3, the characteristics and the key finding of the multicomponent program are presented. Multicomponent interventions were included in all articles. The interventions included consisted of elements of occupational therapy and physical therapy [18], occupational therapy and tai chi exercise [19], memory training and computer-based games [21], cognitive behavioral therapy and qigong exercise [20], multidisciplinary education [22], and cognitive training [23]. The duration of the intervention varied: 6 weeks [21], 8 weeks [23], 23 weeks [22], and 6 months [18,19,20]. The total sessions of intervention were 5 [23], 6 [18,20,21], 10 [19], and up to 32 sessions [22]. Intervention period per session were 60 min [19], 90 min [18], 2 h [21,23], 1 h to 1 h and a half [20], and 15 min to 2 and a half hours [22]. The follow-up period was 4 weeks, 3 months, 6 months, and 12 months from the baseline. Except for one article [19], the remaining five articles were follow-up twice (T1 and T2) from the intervention point (baseline) [18,20,21,22,23].

### 3.4. The Effectiveness of Multicomponent Interventions

ADL and IADL positively and significantly increased in three articles and were maintained over time [19,20,21]. However, in the article of Farzin et al. [21], there was a significant effect not only in the experimental group but also in the control group. In one article, ADL and IADL were significantly increased and maintained up to 6 months (T1) but were not maintained at 12 months (T2) [18]. In one article, ADL had no significant improvement effect, but IADL was positively and significantly improved and maintained over time [22]. In the one remaining article, the intervention had no effect [23]. There were no articles reporting adverse events as a result of the intervention.

## 4. Discussion

This systematic review analyzed the characteristics of multicomponent interventions applied to the community-dwelling elderly over the past 20 years, and it identified their effects on ADL and IADL. Based on the results of this study, there are potential strengths that can lay the foundation for effective interventions for the health promotion and the successful aging process of the community-dwelling elderly in the future. Six articles met the requirements of the inclusion criteria, and all articles were reviewed. Overall, the results of this study suggest that multicomponent interventions can positively affect the ADL and IADL of the community-dwelling elderly. Multicomponent interventions were confirmed to have a positive effect in four articles [18,19,20,22], except for two articles [21,23].

Together with the results of a previous systematic review [24], the results of this review provided additional evidence that multicomponent interventions are effective in promoting ADL and IADL in the community-dwelling elderly. The study of Daniels et al. [24] consisted of studies that were published up to May 2007, including frail elderly living in the community. Also, it included body-related multicomponent interventions with a focus on indicators of body weakness. In contrast, this systematic review analyzed the components and the effects of a multicomponent intervention without focusing on only one indicator for the literature published that were between January 2000 to December 2020. The six selected articles were published in the past 16 years. Considering that four articles have been published in the last four years, it can be seen that researchers’ interest has been focused on this field. The theoretical background for intervention design was mentioned in three out of the six articles [18,19,22], but it was not considered—although its importance has been emphasized in intervention development. In this review, we could not conclude that the literature to which the theoretical background was applied obtained better outcomes. Nevertheless, theories and frameworks improve study outcomes by increasing the possibility of establishing evidence-based interventions [25], and they can secure the justification that research should be conducted based on logically developed concepts and premises [26]. Interventions should be developed based on a well-defined framework given the complexity in the behavioral change of participants, which may increase rigor during the study [27]. Therefore, in future intervention studies, the study design should be established under logical and scientific grounds based on the theoretical background. Additionally, when considering the analysis of study outcomes, if variables are configured to be standardized by a framework, comparability between variables will be easy. The number of samples varied from 12 to 160 per group, and three articles did not mention the rationale for calculating the sample size [19,20,23]. Depending on the sample size, the effectiveness of some interventions may show false positives, so the sample size should be determined according to the rationale for calculation. Although there was no literature mentioning health information literacy, the health information literacy of each elderly person may be different. Accordingly, considering the health information literacy of the participants, their literacy skills should be pre-evaluated to enable them to obtain, understand, evaluate, and make decisions on issues that may affect their health status [28]. There was only one article describing a measurement instrument whose reliability and validity were verified [18], and the rest of the articles did not mention the psychometric properties of the measurement instrument [19,20,21,22,23]. For high-quality study outcomes, it is necessary to provide a basis for an in-depth evaluation on the attributes of how all aspects of the instrument were approached [29]. Therefore, it is necessary for researchers to carefully select and to mention appropriate and accurate instruments to ensure the quality of study outcomes. As such, since the methodological quality of each selected literature is different, it should be interpreted with caution when analyzing the rationale for the intervention.

The components of multicomponent intervention consisted of occupational therapy, physical therapy, exercise, memory training, computer-based games, cognitive behavioral therapy, cognitive training, and multidisciplinary education. In the literature, including occupational therapy as part of multicomponent interventions, studies have reported that it is effective in improving ADL and IADL [18,19]. These results support the strength of occupational therapy in occupation-oriented exercise, including physical activity, to improve ADL in the elderly living in the community [30]. In particular, the application of home visits or home-based occupational therapy interventions can ameliorate the difficulties of ADL [30]. In two articles, exercises, such as tai chi and paldangeum qigong, were included [19,20], and in three articles, physical activities related to ADL were included [18,21,22]. As a result, they were found to be effective in improving ADL and IADL. In contrast, one article that provided a multicomponent cognitive training intervention did not include physical activity and exercise, and it did not produce an improvement effect [23]. As such, exercise and physical activity that maintain the functional status of the elderly clearly showed the strength of effectively improving ADL and IADL [31,32,33,34]. Considering that physical activity intervention has a significant improvement effect on movement and ADL in the elderly, and the more active the elderly, the greater the improvement [34], it is necessary to develop a program that includes ADL and IADL training as a component of the multicomponent intervention.

The multicomponent cognitive training intervention, which had no effect on IADL improvement, consisted of the domains of executive function, attention, attention and memory, memory, and spatiotemporal function [23]. Interestingly, this was in contrast to previous studies in which advanced cognitive training was effective in improving the difficulty of IADL [35]. Moreover, considering that the duration of intervention in other articles was 6 months, the 8 weeks of intervention may be too short in determining the effect on behavioral changes in the elderly. Interestingly, as 8 weeks of cognitive training in the elderly without cognitive function problems was effective in improving IADL [36], the 8 weeks of intervention should be supported by consistent evidence through future studies. However, it is clear that IADL in the elderly cannot be addressed by memory and cognitive training interventions alone [36]. As such, if it is considered to include the components of physical activity in ADL training and drug education in IADL [12,34,37], it is thought to have a positive effect on both ADL and IADL.

Several limitations of this review should be noted. First, the duration of intervention in four articles was 6 months, whereas it was 6 weeks and 8 weeks in two articles. This duration may be too short to understand the impact of the results. Such short durations of intervention may make it impossible to measure the long-term effects on ADL. Therefore, it is important for future studies to evaluate the effectiveness of interventions for short-term and long-term applications. Second, a pilot study in which the rationale for calculating the sample size was not mentioned was included. Since some effects may show false positives depending on the sample size, future studies using the sample size calculated based on the rationale must be conducted. Third, only articles published in English were selected. Therefore, there is a publication bias, as articles published in non-English languages are not included.

## 5. Conclusions

In conclusion, the results of this review suggest the positive outcomes of 6-month multicomponent intervention studies that aim to improve ADL and IADL in the elderly living in the community. Considering the physical condition of the elderly, multicomponent interventions, including physical activity, exercise, occupational therapy, and especially individualized coaching related to ADL and IADL training may be useful. For high-quality studies, along with the selection of standardized measurement instruments, a framework for designing multicomponent interventions should be developed to improve ADL and IADL effectively.

## Figures and Tables

**Table 1:** Methodological study characteristics.

Author	Country of Origin	Study Design	Theoretical Background	Evidence of Sample Size	Sample Characteristics		Measurements
					Intervention Group	Control Group	
Gitlin et al. [18]	USA	RCT	life span theory	yes	n = 160 age: M ± SD 79.5 ± 6.1	n = 159 age: M ± SD 78.5 ± 5.7	ADL difficulty IADL difficulty Fear of falling Confidence performing daily tasks Use of adaptive strategies
Szanton et al. [19]	USA	RCT	extrinsic and intrinsic theory	no	n = 24 age: M ± SD 79 ± 8.2	n = 16 age: M ± SD 77 ± 7.1	ADL difficulty IADL difficulty Health-related quality of life Falls efficacy
Farzin et al. [21]	Malaysia	RCT	no	yes	n = 13 age: M ± SD 63.7 ± 4.8	n = 12 age: M ± SD 62.9 ± 4.1	IADL Depression Anxiety
Jing et al. [20]	China	RCT	no	no	n = 39 age: M ± SD 74.7 ± 6.1	A group: n = 40 age: M ± SD 75.1 ± 5.2 B group: n = 39 age: M ± SD 75.3 ± 6.8	Spirometry ADL dysfunction Health Loneliness Depression
van Lieshout et al. [22]	Netherland	RCT	theoretical framework of frailty	yes	n = 139 age: M ± SD 73.3 ± 6.7	n = 142 age: M ± SD 74.7 ± 7.6	ADL Quality of life IADL Physical fitness (hand grip strength) Functional capacity Walking speed Mobility Depression Loneliness Nutritional status
Srisuwan et al. [23]	Thailand	RCT	no	no	n = 160 age: M ± SD 79.5 ± 6.1	n = 159 age: M ± SD 78.5 ± 5.7	Cognitive function Anxiety Depression IADL

Abbreviations: RCT, randomized controlled trial; M, mean; SD, standard deviation; ADL, activities of daily living; IADL, instrumental activities of daily living.

**Table 2:** Methodological quality of the studies, assessed according to the Cochrane Collaboration tool.

Author	Random Sequence Generation	Allocation Concealment	Blinding of Participants and Researchers	Blinding of Outcome Assessment	Incomplete Outcome Data	Selective Reporting	Other Bias
Gitlin et al. [18]	↓	↓	↓	↓	↓	↓	…
Szanton et al. [19]	…	…	↓	↑	↓	↓	↓
Farzin et al. [21]	↓	↓	↓	↓	↓	↓	↓
Jing et al. [20]	…	…	…	↓	↓	↓	…
van Lieshout et al. [22]	↓	↑	↑	↓	↓	↓	↓
Srisuwan et al. [23]	…	…	↓	↓	…	↓	↓

Abbreviations: ↓, low risk of bias; ↑, high risk of bias; …, unclear risk of bias.

**Table 3:** Characteristics of the multicomponent programs.

Author and Year	Intervention Components	Duration of Study	Procedures	Time Points of Measurements	Results
Gitlin et al. [18]	Occupational and Physical Therapy Education and problem solving Home modification Energy conserving techniques Balance, muscle strengthening Fall-recovery techniques	6 months	Occupational Therapy Five occupational therapy contacts: four 90-min visits and one 20-min telephone contact Physical Therapy: One physical therapy visit (90 min)	Baseline 6 months (T1) 12 months (T2)	At 6 months, intervention participants had less difficulty with IADLs (*p* = 0.03) and ADL (*p* = 0.04) than controls, with largest benefits occurring in bathing (*p* = 0.02) and toileting (*p* = 0.049).
Szanton et al. [19]	CAPABLE (Community Aging in Place, Advancing Better Living for Elders) Occupational Therapy Extrinsic: Housing safety Intrinsic: Individual factor: Self-care, Communication with PCP (primary care provider), and Medication Management Physiological factors: Strength/balance (exercise; Tai chi), Depression, and Pain	6 months	10 in-home sessions, each 60 min duration	Baseline 6 months (T1)	The intervention group improved on all outcomes.
Farzin et al. [21]	Multicomponent Prospective Memory Training Program Strategy-based activities: Daily living PM (Prospective Memory) tasks Process-based training program: Computer-based board game (Virtual Week Board Game)	6 weeks	1 session per week 2 h per session	Baseline 4-week (T1) 12-week (T2)	Statistically significant improvements in the level of IADL among all participants (*p* < 0.05).
Jing et al. [20]	Cognitive–behavioral therapy (CBT) and Baduanjin Qigong CBT: To eliminate negative emotions and behaviors Baduanjin Qigong: Physical activity, Breathing regulation, and Psychological adjustment)	6 months	Every 15 days over the first 3 months, totaling 6 times Each visit lasted 1 to 1.5 h	Baseline 3 months (T1) 6 months (T2)	ADL dysfunction was significantly lowered (*p* < 0.05) in the group receiving joint Baduanjin and CBT intervention at 3 months and 6 months, as compared to the Baduanjin only group or the CBT only group.
van Lieshout et al. [22]	SPRY (Supporting PRoactive lifestyle) multicomponent interdisciplinary intervention Optimization of medication use: Medication review using the Prescribing Optimization Method (POM) Physical fitness improvement: Walking stairs, Shopping, Moving outdoors, and Standing up from a chair or a bed Empowerment of social skills: Assertiveness, Communication styles, Asking for and giving help, Self-appreciation, Saying ‘no’, Giving one’s opinion, and Making plans for the future Improvement of nutritional status: Healthy food, Healthy food in relation to advancing age, Overweight and underweight, and Consequences of bad nutritional status	23 weeks	Medication review: 15 to 45 min Physical fitness: 12 weeks with two one-hour meetings each week Social skills: five meetings once a week for approximately 2.5 h Nutrition: 2.5 h up to three times	Baseline 6 months (T1) 12 months (T2)	After the 12-month follow-up, the ADL score did not change significantly between the two groups. IADL improved significantly after the one-year follow-up (*p* = 0.04).
Srisuwan et al. [23]	CT (cognitive training) TEAM-V (training of executive functions, attention, memory, and visuospatial functions) Program Executive function: Management skills Attention: Switching, selective and sustained attentions Attention and memory: Attention and short-term memory Memory: Short and long-term memory Visuospatial: Spatial-temporal reasoning	8 weeks	Five sessions, with a 2-week interval between each session and 120 min per session	Baseline 6 months (T1) 12 months (T2)	No significant differences were found in neuropsychological and IADL assessment results between two groups at 1 year.

Abbreviations: T1, first follow-up; T2, second follow-up.

## Data Availability

No new data were created or analyzed in this study. Data sharing is not applicable to this article.
